# Analgesia and sedation increases bronchopulmonary dysplasia risk without significant neurodevelopmental impairment in very preterm infants

**DOI:** 10.3389/fmed.2025.1694251

**Published:** 2025-11-28

**Authors:** Bowen Weng, Chongbing Yan, Yuanyang Zhang, Yibo Liu, Cheng Cai

**Affiliations:** Department of Neonatology, Shanghai Children’s Hospital, School of Medicine, Shanghai Jiao Tong University, Shanghai, China

**Keywords:** preterm infant, analgesia, sedation, bronchopulmonary dysplasia, neurodevelopment

## Abstract

**Background:**

The associations between analgesia and/or sedation exposure and adverse outcomes in very preterm infants remain controversial. Evidence linking these medications to moderate-to-severe bronchopulmonary dysplasia (BPD) and long-term neurodevelopmental outcomes is particularly limited in large Chinese preterm cohorts.

**Objective:**

The primary aim was to investigate the associations between exposure to analgesia/sedation and the composite outcome of BPD or death. The secondary aim was to assess the association with neurodevelopmental outcomes at 18 months.

**Methods:**

This large, retrospective cohort study included preterm infants (gestational age < 32 weeks) admitted to a tertiary NICU in Shanghai, China, from 2018 to 2022. The primary outcomes were two composite measures: (1) moderate-to-severe BPD or death, and (2) any BPD or death, both assessed at 36 weeks postmenstrual age. The secondary outcomes included neurodevelopmental outcomes at 18 months and key neonatal morbidities. Propensity score matching (PSM) followed by multivariable logistic regression was used to adjust for confounders.

**Results:**

Of the 713 infants included in the primary analysis, 116 (16.3%) were exposed to analgesia/sedation. In the PSM cohort (*n* = 460), after multivariable adjustment, exposure was associated with a significantly increased risk of moderate-to-severe BPD or death (adjusted *OR*, 6.00; 95% *CI*, 3.35–10.74; *p* < 0.001) and any BPD or death (adjusted *OR*, 5.32; 95% *CI*, 3.15–8.98; *p* < 0.001). Furthermore, the exposure group experienced significantly longer durations of invasive mechanical ventilation (IMV), higher incidences of hypotension requiring intervention and feeding intolerance (all *p* < 0.05). In a separate analysis of the matched neurodevelopmental subcohort (*n* = 129), no significant associations were found with neurodevelopmental outcomes at 18 months.

**Conclusion:**

In this large Chinese preterm cohort, analgesia/sedation exposure was significantly associated with an increased risk of BPD or death. This association was observed alongside a series of neonatal morbidities such as prolonged IMV and hemodynamic instability, though no significant long-term neurodevelopmental harm was detected at 18 months.

## 1 Introduction

Preterm infants in the neonatal intensive care unit (NICU) are frequently exposed to numerous painful and stressful procedures, such as invasive mechanical ventilation (IMV) and other medical interventions (1). There is growing evidence that repeated pain and stress in early life can adversely affect brain development and long-term outcomes (1, 2). On the other hand, analgesia and sedation may cause side effects such as prolonged IMV, hypotension, gastrointestinal complications, and potential long-term neurodevelopmental impairment (3). This presents clinicians with a significant dilemma: how to balance the necessity of providing adequate analgesia against the potential risks of the medications.

This challenge is compounded by conflicting evidence regarding the safety and efficacy of commonly used agents (4, 5). Fentanyl, a primary opioid in our center and many NICUs, is a case in point (6). Studies of its impact on bronchopulmonary dysplasia (BPD) and long-term neurodevelopment in preterm infants less than 32 weeks of gestational age (GA) have shown inconsistent results (7, 8).

This uncertainty contributes to the wide variations in clinical practice observed globally, despite international pain management guidelines (9, 10). This knowledge-to-practice gap is particularly prominent in some regions. For example, a large-scale survey in China revealed that while a majority of neonates suffer from pain, approximately half of NICUs have not yet implemented routine pain management (10). Furthermore, long-term neurodevelopmental data on the impact of these medications in Chinese preterm infants are virtually absent, posing a significant challenge to clinical decision-making in the country. This highlights the critical need for large, real-world studies to clarify these risks.

To address this critical evidence gap, our study aimed to investigate the associations between analgesia/sedation exposure and two key outcomes: moderate-to-severe BPD or death, and long-term neurodevelopment. Our analysis was conducted in a large, real-world cohort of preterm infants (GA < 32 weeks), using a two-stage statistical adjustment strategy.

## 2 Methods

### 2.1 Study design and participants

This retrospective cohort study was conducted at the NICU of Shanghai Children’s Hospital, a tertiary referral center. We included infants who met the following criteria: (1) GA < 32 weeks and (2) admission to our NICU between January 1, 2018, and December 31, 2022. Infants were excluded if they: (1) had severe congenital abnormalities; (2) were admitted at > 14 days of age; (3) had repeated admissions; (4) died within 14 days of birth; or (5) were discharged against medical advice (AMA) within 14 days. We implemented an early 14-day cutoff for exclusions to ensure the final cohort consisted of infants with comparable and complete clinical data. This strategy was critical for minimizing potential bias from unknown clinical histories or incomplete outcome assessments.

**Figure 1:**
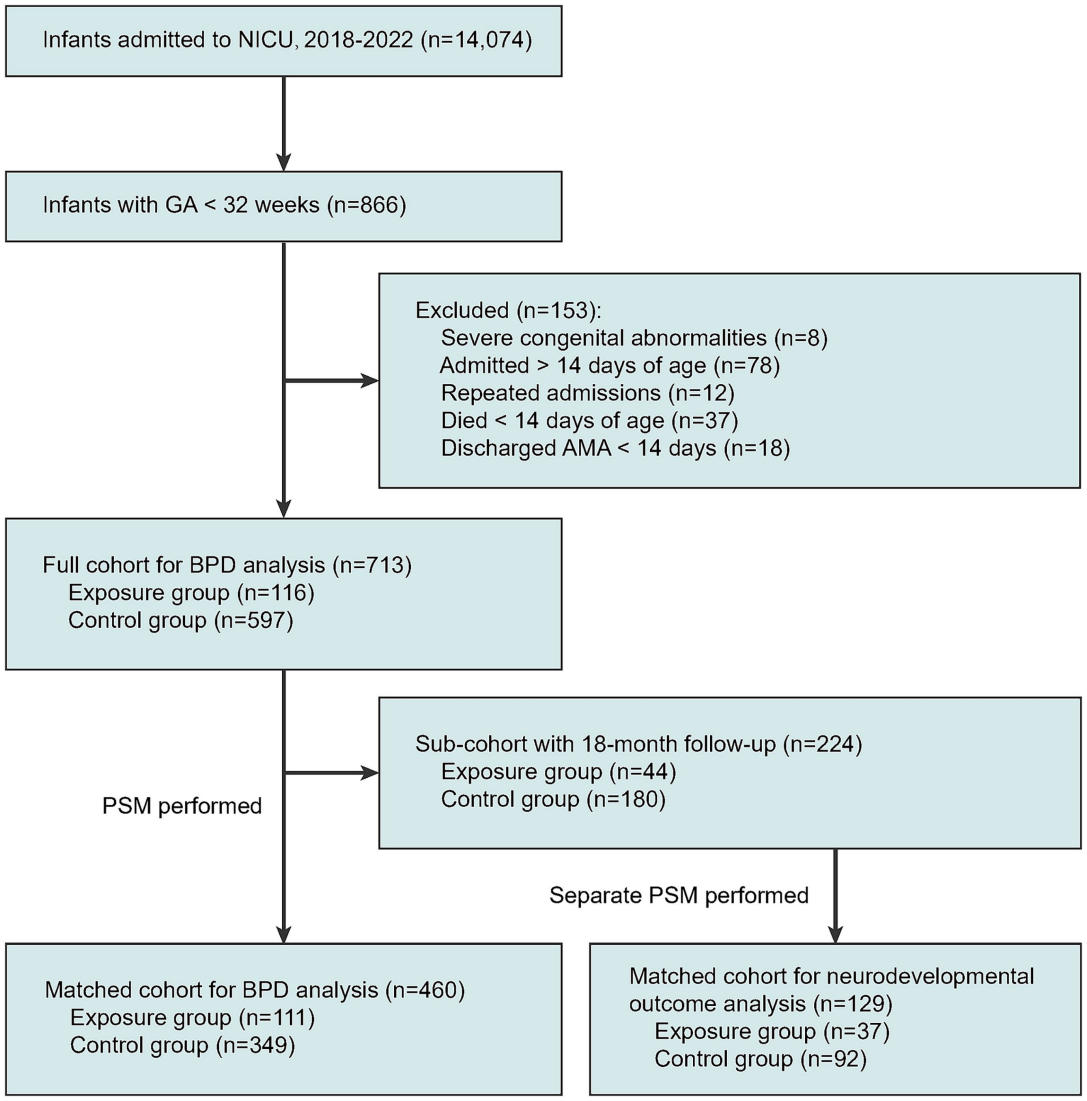
Flowchart of the study population. AMA, against medical advice; BPD, bronchopulmonary dysplasia; GA, gestational age; NICU, neonatal intensive care unit; PSM, propensity score matching.

As detailed in the patient selection flowchart (Figure 1), of the 14,074 infants admitted during the study period, 866 had a GA < 32 weeks. After excluding 153 infants, a final cohort of 713 infants was eligible for the primary BPD analysis. A subcohort of 224 infants with available 18-month follow-up data was analyzed for neurodevelopmental outcomes. Propensity score matching (PSM) was performed separately for both the BPD and neurodevelopmental analyses to balance baseline characteristics.

This study was approved by the Ethics Committee of Shanghai Children’s Hospital (approval number: 2024R014-F01). The requirement for individual informed consent was waived because of the retrospective nature of the analysis.

### 2.2 Data collection

All data on maternal and infant characteristics, clinical course, and outcomes were retrospectively collected from the electronic medical records.

### 2.3 Exposure and group assignment

For this study, exposure to analgesia/sedation was specifically defined as receiving fentanyl and/or midazolam for nonprocedural purposes for more than 24 consecutive hours during the NICU stay. This threshold is consistent with methodologies used in critical care research to evaluate the effects of sustained analgesia/sedation exposure (11–13). Defining exposure in this manner allows for a focused analysis on the potential consequences specifically associated with prolonged continuous infusion (14). In our center, the primary indication for these sustained infusions was to manage irritability or patient-ventilator asynchrony during IMV. The infants who met this criterion formed the exposure group, while those who did not receive any nonprocedural fentanyl or midazolam, or who received it for less than 24 consecutive hours, formed the control group.

### 2.4 Key covariate definitions

The following key covariates were defined: (1) Prolonged premature rupture of the membranes (PPROM) was defined as membrane rupture that occurred 18 h or more prior to delivery. (2) Fetal growth restriction (FGR) was defined as an estimated fetal weight or abdominal circumference below the 10th percentile for GA (15). (3) Intensive resuscitation was defined as the need for positive pressure ventilation, intubation, chest compressions, or epinephrine administration during delivery room resuscitation.

### 2.5 Primary outcomes

The study had two primary outcomes, both assessed at postmenstrual age (PMA) 36 weeks: (1) a composite of moderate-to-severe BPD or death, and (2) a composite of any BPD or death. BPD severity was graded according to the 2018 National Institutes of Health workshop criteria (16). These BPD-related outcomes were assessed in the full analysis cohort (*n* = 713) and its matched subset (*n* = 460).

### 2.6 Secondary outcomes

The secondary outcomes included neurodevelopmental outcomes and several key neonatal morbidities.

The neurodevelopmental outcomes were assessed at a corrected age of 16–20 months by certified examiners using the Gesell Developmental Schedules (GDS). The GDS includes five domains: gross motor, fine motor, adaptive behavior, language, and social behavior. The developmental quotient (DQ) for each domain was calculated on the basis of the infants’ developmental age relative to their corrected chronological age. We analyzed two DQ score thresholds: (1) At risk for neurodevelopmental concerns, defined as a DQ score < 85 [>1 standard deviation (*SD*) below the mean], which is commonly used to identify infants requiring closer monitoring or early intervention. (2) Significant developmental delay, defined as a DQ score < 70 (>2 *SD*s below the mean). While the precise GDS cutoff for a formal neurodevelopmental impairment (NDI) diagnosis is debated, this threshold is an internationally accepted marker for clinically significant delay that warrants therapeutic intervention. This neurodevelopmental analysis was performed exclusively in the subcohort of infants with available follow-up data (*n* = 224) and its matched subset (*n* = 129).

Other secondary outcomes, assessed in the full analysis cohort (*n* = 713) and its matched subset (*n* = 460), were defined as follows: (1) duration of IMV and duration of oxygen therapy; (2) ventilator-associated pneumonia (VAP): a nosocomial lung infection diagnosed in patients who had been receiving IMV for at least 48 h; (3) intraventricular hemorrhage (IVH): defined as grade ≥ 3 according to the Papile criteria; (4) periventricular leukomalacia (PVL): the presence of periventricular cysts identified by either cranial ultrasonography or magnetic resonance imaging; (5) hypotension requiring intervention: hypotension in infants who needed fluid resuscitation or vasoactive drugs; (6) feeding intolerance: defined as the interruption of enteral feeds due to high gastric residual volume, abdominal distention, vomiting or diarrhea; (7) necrotizing enterocolitis (NEC): stage 2 or higher according to the modified Bell staging criteria; and (8) extrauterine growth restriction (EUGR): a decline in the weight-for-age Z score of more than 1.0 from birth to discharge.

### 2.7 Statistical analysis

Continuous variables were tested for normality via the Shapiro–Wilk test. Data are presented as the median (interquartile range, *IQR*) for nonnormally distributed variables and *n* (%) for categorical variables. Group comparisons were performed via the Mann–Whitney U test for continuous variables and the Pearson’s chi–square test or Fisher’s exact test for categorical variables.

To minimize selection bias, two separate propensity score matching (PSM) analyses were performed. For the BPD analysis, nearest-neighbor matching with a variable ratio of up to 1:4 and a caliper of 0.2 was performed without replacement on the full cohort (*n* = 713), yielding a matched cohort of 460 infants. For the neurodevelopmental analysis, a separate PSM with a variable ratio of up to 1:3 and a 0.2 caliper was performed on the subcohort with follow-up data (*n* = 224), yielding a matched subcohort of 129 infants. We chose different matching ratios for each analysis to optimize the balance between statistical power and match quality. This approach was based on the specific sample sizes and exposure-to-control proportions within each cohort. The balance of covariates after matching was assessed via the absolute standardized mean difference (*SMD*), with a value < 0.1 considered a negligible imbalance.

Binary logistic regression was used to calculate the crude and adjusted odds ratios (*OR*s) with 95% confidence intervals (*CI*s). To account for residual confounding after matching, multivariable models were constructed. The selection of covariates for the models was based on two criteria: (1) covariates that remained imbalanced after matching (*SMD* > 0.1), and (2) other clinically critical confounders [e.g., GA, birth weight (BW), sex]. This selection was constrained by the need to maintain an adequate number of events per variable to ensure model stability. Multicollinearity was assessed by calculating the variance inflation factor (VIF), with all the VIF values being < 2.0, indicating no significant collinearity. An exploratory analysis was also performed by further adjusting for the duration of IMV in the BPD cohort. For the outcome of a DQ score < 70, multivariable adjustment was not performed due to the limited number of events to prevent model overfitting.

A two-sided *p* value < 0.05 was considered statistically significant. All analyses were conducted via SPSS Statistics software, version 23 (IBM, USA).

## 3 Results

### 3.1 Study population and baseline characteristics

The patient selection process is detailed in Figure 1. Among the 14,074 infants admitted to our NICU between 2018 and 2022, 866 had a GA of less than 32 weeks. After 153 infants were excluded according to predefined criteria, a final cohort of 713 infants was included for the primary analysis of BPD. Among these, a subcohort of 224 infants with available 18-month follow-up data was identified for the neurodevelopmental outcome analysis.

The baseline characteristics of the full cohort (*n* = 713) are presented in Table 1. The cohort was characterized by a median GA of 30.1 weeks (*IQR*, 29.0–31.1) and a median BW of 1380.0 g (*IQR*, 1162.5–1600.0), with 400 (56.1%) males. Overall, 116 (16.3%) infants received analgesia/sedation (the exposure group), whereas 597 (83.7%) did not (the control group). Specifically, fentanyl was the primary agent used; 13 (1.8%) infants received both fentanyl and midazolam, while none received midazolam alone.

**Table 1:** Baseline characteristics of the study cohort before propensity score matching (*n* = 713).

Characteristic	Exposure group (*n* = 116)	Control group (*n* = 597)	*p* value
Infant characteristics			
Male, *n* (%)	79 (68.1)	321 (53.8)	**0.004**
Gestational age, median (*IQR*), wk	29.4 (28.0, 30.6)	30.3 (29.1, 31.3)	**< 0.001**
Birth Weight, median (*IQR*), g	1252.5 (1026.3, 1455.0)	1405.0 (1200.0, 1612.5)	**< 0.001**
5-min Apgar score, median (*IQR*)	8 (8, 9)	9 (8, 9)	**0.001**
Intensive resuscitation, *n* (%)	61 (52.6)	164 (27.5)	**< 0.001**
Maternal and delivery history			
IVF, *n* (%)	28 (24.1)	172 (28.8)	0.305
Gestational hypertension, *n* (%)	21 (18.1)	95 (15.9)	0.559
GDM, *n* (%)	15 (12.9)	85 (14.2)	0.711
PPROM, *n* (%)	30 (25.9)	133 (22.3)	0.400
FGR, *n* (%)	7 (6.0)	23 (3.9)	0.284
Antenatal antibiotics, *n* (%)	37 (31.9)	201 (33.7)	0.711
Antenatal corticosteroid, *n* (%)	74 (63.8)	421 (70.5)	0.150
Antenatal magnesium sulfate, *n* (%)	55 (47.4)	369 (61.8)	**0.004**
Cesarean section, *n* (%)	73 (62.9)	383 (64.2)	0.802

Data are presented as the *n* (%) or median (IQR). Significant *p* values (< 0.05) are bolded.

IQR, interquartile range; IVF, in vitro fertilization; GDM, gestational diabetes mellitus; PPROM, prolonged premature rupture of the membrane; FGR, fetal growth restriction.

As shown in Table 1, significant baseline differences were observed between the two groups. Compared with those in the control group, infants in the exposure group were more likely to be male and had significantly lower GA, BW, and 5-min Apgar scores. Furthermore, these patients were more likely to require intensive resuscitation. Most maternal characteristics and the mode of delivery did not differ significantly between the groups, with the exception of antenatal magnesium sulfate use.

### 3.2 Primary outcome: association with BPD-related composite outcomes

In an initial analysis of the full, unmatched cohort (*n* = 713), a strong crude association was found between analgesia/sedation exposure and the composite outcome of any BPD or death (*OR*, 6.72; 95% *CI*, 4.39–10.30; *p* < 0.001). To address the significant baseline imbalances detailed in Table 1, PSM was performed. This process yielded a matched cohort of 460 infants, comprising 111 in the exposure group and 349 in the control group. The baseline covariates were substantially more balanced after PSM (Supplementary Table S1). Although no statistically significant differences were detected on the basis of *p* values, some residual imbalance remained. Specifically, several key variables still had an absolute *SMD* > 0.1, including GA, BW, the need for intensive resuscitation, and antenatal magnesium sulfate use.

**Figure 2:**
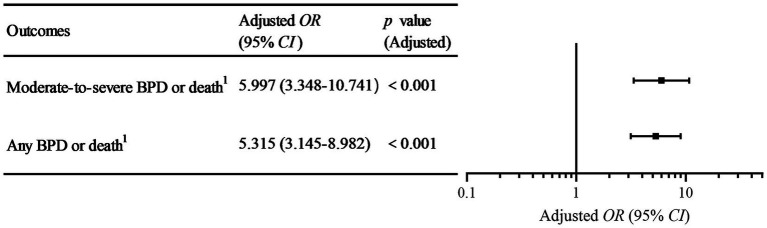
Forest plot for the association between analgesia/sedation exposure and BPD-related composite outcomes. ^1^Death was defined as all-cause mortality before 36 weeks postmenstrual age. The plot displays the adjusted *OR*s and their 95% *CI*s derived from the multivariable logistic regression analysis performed on the propensity score-matched cohort (*n* = 460). The squares represent the point estimate of the a*OR*, and the horizontal lines represent the 95% *CI.* BPD, bronchopulmonary dysplasia; *OR*, odds ratio; *CI*, confidence interval.

The associations between analgesia/sedation exposure and BPD-related composite outcomes were then reassessed within this matched cohort of 460 infants. The results are detailed in Table 2 and visualized in Figure 2. In the unadjusted analysis, the associations remained highly significant for both moderate-to-severe BPD or death (Crude *OR*, 5.64; 95% *CI*, 3.35–9.50; *p* < 0.001) and any BPD or death (Crude *OR*, 4.87; 95% *CI*, 3.09–7.68; *p* < 0.001).

**Table 2:** Associations between analgesia/sedation exposure and BPD-related composite outcomes in the matched cohort (*n* = 460).

Primary outcome	Exposure group (*n* = 111) *n* (%)	Control group (*n* = 349) *n* (%)	Crude *OR* (95% *CI*)	*p* value (Unadjusted)	Adjusted *OR* (95% *CI*)^1^	*p* value (Adjusted)
Moderate-to-severe BPD or death^2^	42 (37.8)	34 (9.7)	5.639 (3.347, 9.503)	**< 0.001**	5.997 (3.348–10.741)	**< 0.001**
Any BPD or death^2^	62 (55.9)	72 (20.6)	4.868 (3.087, 7.677)	**< 0.001**	5.315 (3.145–8.982)	**< 0.001**

^1^Adjusted for gestational age, birth weight, need for intensive resuscitation, antenatal magnesium sulfate exposure, and sex.

^2^Death was defined as all-cause mortality before 36 weeks postmenstrual age.

Significant *p* values (< 0.05) are bolded.

BPD, bronchopulmonary dysplasia; OR, odds ratio; CI, confidence interval.

To account for the residual confounding indicated by the *SMD* analysis, a final multivariable logistic regression model was constructed. The model was adjusted for sex and the covariates that remained imbalanced after matching. Collinearity was assessed and was not a concern (all VIFs < 2.0). In this multivariable adjusted model, exposure to analgesia/sedation remained significantly associated with an increased risk for both moderate-to-severe BPD or death (adjusted *OR*, 6.00; 95% *CI*, 3.35–10.74; *p* < 0.001) and any BPD or death (adjusted *OR*, 5.32; 95% *CI*, 3.15–8.98; *p* < 0.001). The full results of the primary multivariable model are presented in Supplementary Table S2.

In an exploratory analysis, we also constructed a multivariable model that included the duration of IMV. In this model, this association remained statistically significant for the primary outcome of moderate-to-severe BPD or death (adjusted *OR*, 2.45; 95% *CI*, 1.19–5.04; *p* = 0.015).

### 3.3 Key secondary outcomes: neurodevelopmental outcomes

The associations with neurodevelopmental outcomes were subsequently assessed in a subcohort of 224 infants with available 18-month follow-up data. As detailed in Supplementary Table S3, this group also exhibited significant baseline imbalances. Therefore, a separate PSM procedure was performed, yielding a new matched subcohort of 129 infants (37 in the exposure group and 92 in the control group) with substantially improved baseline balance (Supplementary Table S4).

The analysis of neurodevelopmental outcomes, performed on this matched subcohort, is detailed in Table 3. For the outcome of a DQ score < 85, no significant associations were found in either the unadjusted analysis (Crude *OR*, 1.31; 95% *CI*, 0.56–3.05; *p* = 0.536) or the multivariable analysis adjusted for residual and clinical confounders (adjusted *OR*, 1.301; 95% *CI*, 0.55–3.07; *p* = 0.548). Similarly, for the more stringent outcome of a DQ score < 70, no significant association was observed in the unadjusted analysis (Crude *OR*, 1.03; 95% *CI*, 0.39–2.73; *p* = 0.954). A multivariable analysis was not performed for this outcome because of the small number of events.

**Table 3:** Associations between analgesia/sedation exposure and neurodevelopmental outcomes in the matched subcohort (*n* = 129).

Key secondary outcomes	Exposure group (*n* = 37) *n* (%)	Control group (*n* = 92) *n* (%)	Crude *OR* (95% *CI*)	*p* value (Unadjusted)	Adjusted *OR* (95% *CI*)^1^	*p* value (Adjusted)
At-risk for developmental concerns (GDS DQ < 85)	27 (73.0)	62 (67.4)	1.306 (0.560, 3.046)	0.536	1.301 (0.551–3.071)	0.548
Significant developmental delay (GDS DQ < 70)	7 (18.9)	17 (18.5)	1.029 (0.388, 2.734)	0.954	NA^2^	NA^2^

^1^Adjusted for in vitro fertilization, cesarean section, need for intensive resuscitation, gestational age, birth weight, and sex.

^2^Multivariable adjustment was not performed for this outcome because of the small number of events.

GDS, Gesell developmental schedules; OR, odds ratio; CI, confidence interval; NA, not applicable.

### 3.4 Other secondary outcomes

A comprehensive analysis of other secondary outcomes was performed on the matched cohort, and the detailed results are presented in Table 4. The infants in the exposure group experienced significantly longer durations of both IMV and oxygen therapy. Furthermore, the incidences of VAP, IVH, hypotension requiring intervention, feeding intolerance, NEC, and EUGR were significantly higher in the exposure group both before and after matching (all *p* < 0.01). In contrast, no significant difference was observed in the incidence of PVL between the two groups after PSM.

**Table 4:** Comparison of other secondary outcomes before and after propensity score matching.

Secondary outcomes	Before PSM			After PSM		
	Exposure group (*n* = 116)	Control group (*n* = 597)	*p* value	Exposure group (*n* = 111)	Control group (*n* = 349)	*p* value
All-cause mortality before discharge, *n* (%)	8 (6.9%)	5 (0.8%)	**< 0.001**	7 (6.3%)	4 (1.1%)	**0.002**
Pulmonary outcomes						
IMV, d, median (*IQR*)	8.5 (5.0, 23.0)	0.0 (0.0, 2.0)	**< 0.001**	8.0 (5.0, 24.0)	0.0 (0.0, 2.0)	**< 0.001**
Oxygen therapy, d, median (*IQR*)	42.0 (19.5, 77.0)	17.0 (7.0, 37.0)	**< 0.001**	42.0 (19.0, 77.0)	26.0 (11.0, 42.0)	**< 0.001**
VAP, *n* (%)	30 (25.9%)	11 (1.8%)	**< 0.001**	26 (23.4%)	10 (2.9%)	**< 0.001**
Neurological outcomes						
Severe IVH (Grade III-IV), *n* (%)	16 (13.8%)	16 (2.7%)	**< 0.001**	16 (14.4%)	13 (3.7%)	**< 0.001**
PVL, *n* (%)	8 (6.9%)	18 (3.0%)	**0.041**	8 (7.2%)	13 (3.7%)	0.126
Circulatory outcome						
Hypotension requiring intervention, *n* (%)	43 (37.1%)	31 (5.2%)	**< 0.001**	41 (36.9%)	19 (5.4%)	**< 0.001**
Gastrointestinal and nutritional						
Feeding intolerance, *n* (%)	95 (81.9%)	369 (61.8%)	**< 0.001**	92 (82.9%)	238 (68.2%)	**0.003**
NEC (≥ Stage II), *n* (%)	15 (12.9%)	15 (2.5%)	**< 0.001**	15 (13.5%)	9 (2.6%)	**< 0.001**
EUGR, *n* (%)	84 (72.4%)	289 (48.4%)	**< 0.001**	81 (73.0%)	188 (53.9%)	**< 0.001**

Data are presented as the median (IQR) or *n* (%). Significant *p* values (< 0.05) are bolded.

PSM, propensity score matching; IQR, interquartile range; IMV, invasive mechanical ventilation; VAP, ventilator-associated pneumonia; IVH, intraventricular hemorrhage; PVL, periventricular leukomalacia; NEC, necrotizing enterocolitis; EUGR, extrauterine growth restriction.

## 4 Discussion

In this large, retrospective cohort study, we found that exposure to analgesia/sedation was significantly associated with an increased risk for BPD or death after statistical adjustment. In contrast, a significant association was not observed for neurodevelopmental outcomes at 18 months of corrected age.

In our real-world study involving 713 preterm infants < 32 weeks, 116 (16.3%) received analgesia/sedation. Wide global practice variation in preterm infant analgesia and sedation reflects concerns about potential adverse effects, despite existing international and national guidelines (10, 17). For example, a survey of 57 tertiary NICUs in China revealed a vast range in the utilization rate of analgesia/sedation among preterm infants with a GA < 32 weeks, from 0 to 72.5% (18). The utilization rate in our cohort (16.3%) is comparable to the overall Chinese average (16.6%) but is notably lower than rates reported in Western countries, such as Canada (29%) and the UK (over 38%) (15, 18, 19). This variability highlights the critical need for region-specific data.

Consistent with other studies, our study found that analgesia/sedation exposure was significantly associated with an increased risk for any BPD or death (adjusted *OR*, 5.32; 95% *CI*, 3.15–8.98; *p* < 0.001) (7, 18, 20). Furthermore, using a two-stage adjustment strategy, we observed that exposure to analgesia/sedation was significantly associated with the composite outcome of moderate-to-severe BPD or death. While our retrospective design cannot draw causal inferences, our findings regarding secondary outcomes allow us to generate hypotheses regarding this association. Our data show that exposure to analgesia/sedation was correlated with a series of adverse events. It is well established that analgesia/sedation can be associated with respiratory depression, which in turn is linked to the prolonged durations of both IMV and oxygen therapy observed in our exposure group (21–23). This extended need for IMV is a known major risk factor for VAP, which was also more frequent in our exposed infants. Second, studies in neonates have shown that analgesia/sedation might be associated with hemodynamic instability, including hypotension and altered cerebral blood flow velocity (3). This is consistent with our findings of a higher incidence of hypotension requiring intervention and IVH, as reported in other cohorts (19). Finally, the well-documented effect of opioids on delaying bowel motility is also consistent with our findings of a higher incidence of gastrointestinal complications and poorer nutritional status in the exposure group (23). Therefore, we hypothesize that these morbidities are not merely concurrent adverse events. Instead, we propose they are key interconnected risk factors within the complex relationship between analgesia/sedation exposure and the risk of BPD or death. This hypothesized complex interrelationship requires validation in future prospective studies.

A critical methodological challenge in this research is the complex relationship between IMV duration and analgesia/sedation exposure. To investigate this, we performed an exploratory analysis by further adjusting for IMV duration in the multivariable model. The association between analgesia/sedation exposure and moderate-to-severe BPD or death, while attenuated (adjusted *OR* decreased from 6.00 to 2.45), remained statistically significant (*p* = 0.015). This finding suggests that prolonged IMV statistically accounts for a large portion of the observed association. We must emphasize the limitations of this model. It is not a formal causal analysis because it cannot untangle the causal direction and is susceptible to over-adjustment bias. While its result (a*OR* 2.45) cannot be interpreted as an independent effect, the persistent statistical significance (*p* = 0.015) suggests the increased risk may not be fully explained by ventilation time alone.

To our knowledge, this is the first study in China to assess long-term neurodevelopmental outcomes in preterm infants (GA < 32 weeks) following neonatal exposure to analgesia/sedation. A particularly interesting finding was the lack of a significant association with the neurodevelopmental outcomes at 18 months, which appears paradoxical given the increased risk of BPD (24, 25). We propose several potential explanations. First, the neuroprotective effect of pain and stress reduction from analgesia/sedation may counteract the well-known negative impact of BPD on neurodevelopment (8). Second, our center is a regional referral hub for critically ill neonates from other districts, making long-term follow-up challenging for many families. Consequently, this analysis was only conducted on a smaller subcohort (*n* = 129), which may have been underpowered to detect a more subtle association (a Type II error). Third, significant survivor bias is likely present. Families who completed the 18-month follow-up may have greater access to early intervention services or higher adherence, which could improve outcomes and mask potential adverse effects (26).

Variations in medication patterns may partly explain why the reported associations between analgesia/sedation exposure and neurodevelopmental outcomes vary across studies (27). For example, a Chinese study reported that the majority (83.1%) of preterm infants received only sedatives, 9.8% received both opioids and sedatives, and 7.1% received only opioids (18). However, evidence suggests that, compared with opioids, midazolam is associated with impaired hippocampal maturation and poorer cognitive outcomes at 18 months (28). Furthermore, prolonged combination therapy with opioids and benzodiazepines is consistently associated with worse neurodevelopmental outcomes, suggesting a superior safety profile for monotherapy (7, 29). While a previous study suggested that short-term morphine may attenuate pain-associated motor deficits (8), another cohort study that assessed brain imaging before discharge revealed an increased risk of preterm brain injury with longer opioid (predominantly morphine) exposure (19). Additionally, a prospective study found no difference in sensorimotor impairments at 2 years in very preterm infants receiving continuous opioids and/or midazolam infusions (30). Given this evidence, the specific pattern of drug use in our center—predominantly fentanyl monotherapy (88.8% of the exposure group)—may explain our finding of no adverse neurodevelopmental impact.

The primary strength of this study is its large, single-center, real-world cohort, which provides robust statistical power while ensuring consistency in clinical practice. Furthermore, our two-stage adjustment strategy, which combines PSM with multivariable regression and is supported by exploratory analyses, offers an estimation of the associations through balancing a wide array of covariates. However, our study has important limitations. First, as a single-center retrospective study, it is susceptible to information bias and limited generalizability. Second, the evolving clinical status of infants could simultaneously drive both the decision to use analgesia/sedation and the final outcome. These dynamically adjusted variables precluded a valid analysis of any dose–response or duration-response relationship. Third, the small number of infants receiving midazolam combination therapy (*n* = 13) limited statistical power to disentangle potential agent-specific effects.

## 5 Conclusion

In conclusion, our real-world study found that analgesia/sedation exposure was significantly associated with an increased risk of BPD or death in preterm infants (GA < 32 weeks). However, no significant long-term neurodevelopmental harm was detected at 18 months. Our findings further showed associations with a series of neonatal morbidities such as prolonged IMV and hemodynamic instability, suggesting these complications are important factors within this complex association. This finding highlights the need for proactive management strategies targeting the respiratory, hemodynamic, and nutritional complications that may arise. Our study calls for future large-scale, prospective research to further investigate these associations and to develop safer, individualized pain and sedation strategies for this vulnerable population.

## Data Availability

The original contributions presented in the study are included in the article/Supplementary material, further inquiries can be directed to the corresponding author/s.
