# Supplementary material for: Analgesia and sedation increases bronchopulmonary dysplasia risk without significant neurodevelopmental impairment in very preterm infants
Source: Front Med (Lausanne). 2025 Nov 28;12:1694251. doi: 10.3389/fmed.2025.1694251 (PMC12698585; doi:10.3389/fmed.2025.1694251)
Supplement: Supplementary file 1 [file Table_1.DOCX]

Table S1 Baseline characteristics of the study cohort after propensity score matching (*n*=460)

| **Characteristic** | **Exposure group**  **(*n*=111)** | **Control group**  **(*n*=349)** | ***p* value** | ***SMD*** |
| --- | --- | --- | --- | --- |
| **Infant Characteristics** |  |  |  |  |
| Male, *n* (%) | 74 (66.7) | 217 (62.2) | 0.095 | 0.095 |
| Gestational age, median (*IQR*), wk | 29.6 (28.1, 30.6) | 29.7 (28.7, 31.0) | 0.115 | **0.193** |
| Birth Weight, median (*IQR*), g | 1260.0 (1047.5, 1477.5) | 1310.0 (1105.0, 1510.0) | 0.155 | **0.137** |
| 5-min Apgar score, median (*IQR*) | 8 (8, 9) | 9 (8, 9) | 0.218 | 0.069 |
| Intensive resuscitation, *n* (%) | 56 (50.5) | 142 (40.7) | 0.070 | **0.195** |
| **Maternal and Delivery History** |  |  |  |  |
| IVF, *n* (%) | 26 (23.4) | 91 (26.1) | 0.576 | 0.063 |
| Gestational hypertension, *n* (%) | 21 (18.9) | 56 (16.1) | 0.499 | 0.073 |
| GDM, *n* (%) | 15 (13.5) | 49 (14.0) | 0.889 | 0.015 |
| PPROM, *n* (%) | 28 (25.2) | 74 (21.2) | 0.789 | 0.093 |
| FGR, *n* (%) | 7 (6.3) | 16 (4.6) | 0.468 | 0.071 |
| Antenatal antibiotics, *n* (%) | 35 (31.5) | 116 (33.2) | 0.739 | 0.037 |
| Antenatal corticosteroid, *n* (%) | 71 (64.0) | 233 (66.8) | 0.588 | 0.058 |
| Antenatal magnesium sulfate, *n* (%) | 55 (49.6) | 193 (55.3) | 0.290 | **0.115** |
| Cesarean section, *n* (%) | 73 (65.8) | 214 (61.3) | 0.399 | 0.094 |

Data are presented as the *n* (%) or median (*IQR*). Significant *p* values (< 0.05) are bolded. An absolute *SMD* > 0.1, indicated in bold, suggests a meaningful imbalance.

Abbreviations: *SMD*, standardized mean difference; *IQR*, interquartile range; IVF, in vitro fertilization; GDM, gestational diabetes mellitus; PPROM, prolonged premature rupture of the membrane; FGR, fetal growth restriction.

Table S2 Full multivariable logistic regression model for risk factors for moderate-to-severe BPD or death in the matched cohort (*n*=460)

| **Variable** | **Adjusted *OR* (95% *CI*)** | ***p* value** |
| --- | --- | --- |
| Analgesia and sedation exposure | 5.997 (3.348, 10.741) | **<0.001** |
| Gestational age | 1.006 (0.976, 1.038) | 0.686 |
| Birth weight | 0.997 (0.995, 0.998) | **<0.001** |
| Intensive resuscitation | 2.174 (1.181, 4.001) | **0.013** |
| Antenatal magnesium sulfate | 1.731 (0.961, 3.118) | 0.068 |
| Sex (male vs female) | 2.089 (1.114, 3.918) | **0.022** |

Significant *p* values (< 0.05) are bolded. Death was defined as all-cause mortality before 36 weeks postmenstrual age.

Abbreviations: BPD, bronchopulmonary dysplasia; *OR*, odds ratio; *CI*, confidence interval.

Table S3 Baseline characteristics of the 18-month follow-up subcohort (*n*=224)

| **Characteristic** | **Exposure group (*n*=44)** | **Control group (*n*=180)** | ***p* value** |
| --- | --- | --- | --- |
| **Infant Characteristics** |  |  |  |
| Male, *n* (%) | 29 (65.9) | 93 (51.7) | 0.089 |
| Gestational age, median (*IQR*), wk | 28.9 (27.5, 30.3) | 30.0 (29.0, 31.1) | **< 0.001** |
| Birth Weight, median (*IQR*), g | 1172.5 (956.3, 1375.0) | 1367.5 (1155.0, 1572.5) | **< 0.001** |
| 5-min Apgar score, median (*IQR*) | 8 (7, 9) | 9 (8, 9) | **0.004** |
| Intensive resuscitation, *n* (%) | 25 (56.8) | 52 (28.9) | **< 0.001** |
| **Maternal and Delivery History** |  |  |  |
| IVF, *n* (%) | 6 (13.6) | 59 (32.8) | **0.012** |
| Gestational hypertension, *n* (%) | 8 (18.2) | 40 (22.2) | 0.558 |
| GDM, *n* (%) | 6 (13.6) | 34 (18.9) | 0.415 |
| PPROM, *n* (%) | 9 (20.5) | 28 (15.6) | 0.433 |
| FGR, *n* (%) | 2 (4.5) | 7 (3.9) | 0.842 |
| Antenatal antibiotics, *n* (%) | 14 (31.8) | 58 (32.2) | 0.959 |
| Antenatal corticosteroid, *n* (%) | 27 (61.4) | 131 (72.8) | 0.137 |
| Antenatal magnesium sulfate, *n* (%) | 22 (50.0) | 119 (66.1) | **0.047** |
| Cesarean section, *n* (%) | 28 (63.6) | 102 (56.7) | 0.401 |

Data are presented as *n* (%) or median (*IQR*); significant *p* values (< 0.05) are bolded.

Abbreviations: *IQR*, interquartile range; IVF, in vitro fertilization; GDM, gestational diabetes mellitus; PPROM, prolonged premature rupture of the membrane; FGR, fetal growth restriction.

Table S4 Baseline characteristics of the 18-month follow-up subcohort after propensity score matching (*n*=129)

| **Characteristic** | **Exposure group  (*n*=37)** | **Control group  (*n*=92)** | ***p* value** | ***SMD*** |
| --- | --- | --- | --- | --- |
| **Infant Characteristics** |  |  |  |  |
| Male, *n* (%) | 24 (64.9) | 58 (63.0) | 0.846 | 0.038 |
| Gestational age, median (*IQR*), wk | 29.1 (28.0, 30.4) | 29.1 (28.1, 30.3) | 0.969 | 0.008 |
| Birth Weight, median (*IQR*), g | 1195.0 (1040.0, 1400.0) | 1242.5 (1013.8, 1376.3) | 0.793 | 0.097 |
| 5-min Apgar score, median (*IQR*) | 8 (8, 9) | 9 (8, 9) | 0.328 | 0.045 |
| Intensive resuscitation, *n* (%) | 19 (51.4) | 41 (44.6) | 0.485 | **0.136** |
| **Maternal and Delivery History** |  |  |  |  |
| IVF, *n* (%) | 5 (13.5) | 16 (17.4) | 0.589 | **0.113** |
| Gestational hypertension, *n* (%) | 8 (21.6) | 22 (23.9) | 0.781 | 0.056 |
| GDM, *n* (%) | 5 (13.5) | 14 (15.2) | 0.805 | 0.050 |
| PPROM, *n* (%) | 6 (16.2) | 17 (18.5) | 0.761 | 0.061 |
| FGR, *n* (%) | 2 (5.4) | 6 (6.5) | 1.000 | 0.049 |
| Antenatal antibiotics, *n* (%) | 10 (27.0) | 27 (29.3) | 0.792 | 0.052 |
| Antenatal corticosteroid, *n* (%) | 24 (64.9) | 63 (68.5) | 0.692 | 0.076 |
| Antenatal magnesium sulfate, *n* (%) | 19 (51.4) | 51 (55.4) | 0.674 | 0.082 |
| Cesarean section, *n* (%) | 25 (67.6) | 57 (62.0) | 0.549 | **0.120** |

Data are presented as the *n* (%) or median (*IQR*). An absolute *SMD* > 0.1, indicated in bold, suggests a meaningful imbalance.

Abbreviations: *SMD*, standardized mean difference; *IQR*, interquartile range; IVF, in vitro fertilization; GDM, gestational diabetes mellitus; PPROM, prolonged premature rupture of the membrane; FGR, fetal growth restriction.
